# Supplementary material for: Are carbon emissions trading and green financial instruments synergistic? -Comprehensive quantitative research based on content analysis
Source: PLoS One. 2024 Mar 7;19(3):e0298601. doi: 10.1371/journal.pone.0298601 (PMC10919601; doi:10.1371/journal.pone.0298601)
Supplement: S1 Data — (ZIP) [file pone.0298601.s001.zip › Data/地级市Z_F工作报告.zip!/╔╜╢1⁄2/╝├─■╩╨/└┤╫╘╣1⁄2╓┌║┼í╛┬φ┐╦╩2╛▌═°í┐_2004.docx]

各位代表：

　　现在，我代表济宁市人民政府向大会报告工作，请予审议，并请市政协委员和其他列席会议的同志提出意见。

　　一、同心同德，奋力拼搏，2003年各项工作取得新成绩

　　刚刚过去的一年极不平凡。国际形势复杂多变，非典疫情突如其来，洪涝灾害多年未遇。面对困难和挑战，全市人民在中共济宁市委的坚强领导下，以邓小平理论和“三个代表”重要思想为指导，认真贯彻党的十六大和十六届三中全会及省、市委工作会议精神，解放思想，干事创业，比学赶超，加快发展，改革开放和现代化建设迈出新步伐。初步核算，全市国内生产总值完成882.6亿元、比上年增长16.7%。财政总收入85.2亿元、增长27.2%，其中地方财政收入47.8亿元、增长26.7%。社会消费品零售总额241.6亿元、增长15.9%。金融机构年末各项存款余额662.8亿元、各项贷款余额492.6亿元，分别比年初增长22.6%和20.7%。完成或超额完成市十四届人大一次会议确定的年度工作任务，全市呈现出经济发展、事业进步、社会安定、政通人和的良好局面。

　　——抗击非典和抗洪救灾取得重大胜利。去年上半年，面对突如其来的非典疫情，各级政府坚决贯彻上级党委、政府的决策部署，全市万众一心，众志成城，迎难而上，严防死守，建立完善“十大防御体系”，狠抓各项防范措施落实，确保了全市人民身体健康、生命安全和经济发展、社会稳定。下半年，部分县乡遭受洪涝灾害，各级领导靠前指挥，有关部门科学调度，军警民并肩作战，社会各界全力救助，及时转移安置灾民，迅速开展生产自救，确保了受灾群众有足够的口粮、御寒的衣被、过冬的住处。

　　——对外经贸大幅增长。成功举办国际孔子文化节、第五届国际儒商大会及中国专利高新技术产品博览会，先后派出918个团组赴国内外集中开展招商活动。全年新批办外商投资项目136个，合同利用外资5.93亿美元、增长131.7%，实际利用外资2.3亿美元、增长160.4%；引进国内合同资金634亿元，实际到位185.2亿元；完成外贸进出口总额10.06亿美元、增长58.7%，其中出口5.3亿美元、增长50%。

　　——改革调整扎实推进。以产权制度改革为突破口，推进国有经济战略性调整，加快建立和完善现代企业制度。农村税费改革成果得到巩固。行政审批制度、住房制度和市政公用事业改革不断深化。立足“调优一产、调高二产、调活三产”，全市三次产业比例由上年的15.6∶46.6∶37.8调整为14.4∶49∶36.6。以“五大产业链”为主线的农业结构调整初见成效。工业经济支撑作用日益增强，全市规模以上工业企业达到769家、新增219家，利税过千万元的达到103家、新增29家，其中过亿元的25家、新增7家。高新技术产业产值占规模以上工业总产值比重达到15.9%，增加4.1个百分点。新兴服务业发展加快，旅游业实现恢复性增长。民营经济增势强劲，完成增加值268.7亿元、增长55.2%。

　　——投资规模持续扩大。全社会固定资产投资达到356亿元、增长52.1%，其中国有及其他单位完成投资215亿元、增长55%。开工建设500万元以上项目1275个，其中生产性项目926个，非生产性项目349个。703个项目竣工运营或部分建成投产。

　　——城市建设又添亮点。济宁高新区基础设施进一步完善，综合实力和服务功能不断增强。中心区“绿亮清”二期工程深入推进，完成投资6.7亿元，新建改造城市道路26条，翻建维修小街巷52条，新增公共绿地37.2万平方米。建成廉租房1万平方米。拆除小锅炉、小烟囱407台（套），集中供热面积由260万平方米扩大到350万平方米，大气异味和烟尘污染治理初见成效，城市空气质量优于国家二级标准的天数由161天增加到280天。主要河道水质好转，部分路段机动车禁鸣，市区人居环境改善。

　　——人民生活水平不断提高。全市城镇居民人均可支配收入8310元、增长15%，农民人均纯收入3178元、增长6.9%。全年新增城镇就业9.4万人，其中下岗失业人员再就业3.3万人，城镇登记失业率3.4%。市县财政投入低保资金3503万元，救助低保对象9.4万人。安排救灾款物1.5亿元，救济受灾群众21万人。硬化农村公路1230公里，开通农村客运线路264条。农村电网改造二期工程竣工。改造农村校舍危房15万平方米。年初承诺为民所办10件实事如期完成。

　　——社会事业全面进步。全年取得重要科技成果152项，申报专利385项。“普九”成果得到巩固，高中阶段扩招新生9347名，济宁学院正在加紧建设。制定完善突发公共卫生事件应急预案，基层医疗机构建设进一步加强，农村改水、改厕初见成效。文化市场健康发展。全民健身活动广泛开展，竞技体育水平提高。完成了省下达的人口与计划生育责任目标，计划生育率保持在98%以上，人口自然增长率为4‰。土地市场治理整顿深入推进，经营性用地招标拍卖和挂牌出让制度全面实行。生态建设、环境保护和资源开发综合效益提高。“双拥共建”工作富有成效，荣获全国双拥模范城“四连冠”。新闻出版、广播电视、外事侨务、民族宗教、地震气象、档案史志、文博以及对台、人防、残疾人、老龄等项事业获得新的发展。

　　——民主政治和精神文明建设日益加强。政府法制建设扎实推进，依法行政水平不断提高。认真执行人大及其常委会各项决议决定，办理人大代表建议203件，政协委员提案358件。廉政建设和反腐败斗争深入开展，全面落实党风廉政建设责任制，强化审计监督和行政监察，进一步规范行政行为，纠正部门和行业不正之风成效明显。广泛开展“四五”普法，积极发展基层民主。深入贯彻《公民道德建设实施纲要》，群众性精神文明创建活动丰富多彩，农村“文明一条街”建设经验在全省推广，一批优秀作品在全省、全国获奖。坚持不懈开展严打整治斗争，完善社会治安防控体系，维护了社会稳定。强化安全生产专项治理，基本杜绝重特大事故发生。

　　各位代表！过去的一年，是我们抗击非典疫情，战胜洪涝灾害，经受严峻考验的一年；是我们牢牢把握“第一要务”，解放思想、干事创业、比学赶超、加快发展，经济建设和社会各项事业扎实推进的一年。一年的成绩来之不易，是省委、省政府和市委坚强领导的结果，是市人大、市政协及社会各界监督支持的结果，是全市人民团结奋进、扎实苦干的结果。在此，我代表市人民政府，向全市工人、农民、知识分子和干部及各界人士，向中国人民解放军驻济部队指战员、武警官兵和公安干警，向中央、省驻济各单位、各企业，向关心支持济宁发展的港澳台同胞、海外侨胞和国际友人，表示衷心的感谢，并致以崇高的敬意！

　　我们也清醒地看到，前进道路上还有不少矛盾、困难和问题。主要是，经济结构不够合理，市场竞争力不强；外经贸规模不大，经济外向度不高；一些领域和行业的改革不彻底、不规范，制约发展的体制性、机制性障碍尚未消除；县域经济发展不平衡，差距有所拉大；农业产业化水平较低，农民增收缓慢；社会保障体系有待完善，就业再就业压力较大；精神文明和民主法制建设存有薄弱环节；人民群众对社会治安、环境保护等工作还有不满意的地方；我们在思想观念和领导方式上也有一些不适应的问题，少数部门服务意识不强、办事效率不高、工作作风不实；发展环境有待进一步优化。对此，必须高度重视，用改革和发展的办法认真加以解决。

　　二、抓住机遇，乘势而上，推动全市经济持续快速协调健康发展和社会全面进步

　　2004年是全面落实党的十六大和十六届三中全会精神、实现“十五”计划目标的关键一年。做好今年政府工作，意义重大而深远。纵观国际国内形势，尽管存在一些不确定因素，但总体上仍然是机遇大于挑战，希望多于困难。全球经济正在回升，国际产业转移与资本扩张加速推进，入世积极效应进一步显现，为我们扩大开放、招商引资开辟了广阔空间；我国正处在经济周期上升阶段，宏观经济政策保持稳定，十六届三中全会在完善社会主义市场经济体制方面实现一系列重大突破，为我们深化改革、创新发展增添了强大动力；我市综合实力进一步增强，一批重大产业项目陆续发挥效益，新签约的合同外资和内资将逐步到位，加之我市得天独厚的煤、电、水资源，为我们扬长避短、竞争发展提供了有力支撑。特别是经过去年复杂环境和各种困难的锻炼考验，各级各部门领导班子驾驭能力和工作水平明显提高，聚精会神搞建设、一心一意谋发展的氛围日益浓厚，为我们比学赶超、加快发展奠定了坚实基础。同时也要看到，国际贸易保护主义呈加剧之势，国家出口退税、信贷投资、土地管理和“三农”等政策的适度调整将带来一些新情况，区域经济竞争更加激烈。因此，我们必须具有不进则退的忧患意识，进一步增强压力感，勇于解决矛盾、克服困难、迎接挑战、经受考验，在攻坚破难中不断前进；必须具有时不我待的机遇意识，进一步增强紧迫感，善于认识机遇、把握机遇、抓住机遇、用好机遇，在激烈竞争中抢占先机；必须具有争先恐后的赶超意识，进一步增强责任感，再掀解放思想、干事创业、比学赶超、加快发展热潮，在奋力拼搏中勇攀高峰，夺取改革开放和现代化建设的新胜利。

　　今年工作的总体要求是：以邓小平理论和“三个代表”重要思想为指导，以全面建设小康社会和建设经济强市、文化名市、组群结构大城市为目标，深入贯彻落实党的十六大及十六届三中全会精神，坚持以人为本、统筹发展，坚持全面、协调、可持续的科学发展观，坚持“全党抓经济，重点抓调整”，坚持举全市之力扩大开放，坚持科教兴市、人才强市战略，坚持工业化与城市化双轮驱动，着力培植外经外贸、民营经济、高新技术产业“三大亮点”，突出抓好各项改革、大项目带动、农业产业化等项重点，更加重视解决“三农”问题，更加重视发展县域经济，更加重视劳动就业和社会保障工作，更加重视政治文明和精神文明建设，促进全市经济持续快速协调健康发展和社会全面进步。经济社会发展的主要预期目标是：国内生产总值增长14%，地方财政收入增长17%，全社会固定资产投资增长35%，规模以上工业增加值、利税、利润均增长20%，外贸进出口增长25%，其中出口增长25%，合同和实际利用外资均增长50%，社会消费品零售总额增长13%，城镇居民人均可支配收入增长9%，农民人均纯收入增长6%，城镇登记失业率控制在4%以内，人口自然增长率控制在6‰以内。

　　三、突出重点，把握关键，增创经济发展新优势

　　实现今年经济工作目标任务，必须突出重点，抓住关键，着力在八个方面下功夫、求突破。

　　（一）继续举全市之力扩大开放，实现外向型经济新突破。按照统筹国内发展和对外开放的总要求，坚持市、县、乡、村四级联动，政府、行业、企业、民间合力推进，产业项目、资源开发、基础设施、社会事业全面招商，领导、政策、环境、考核多措并举，开创济宁扩大开放新局面。

　　健全三个体系。重点健全完善领导责任体系、政策激励体系和社会服务体系。各级各部门主要领导对外经贸工作要亲自抓，分管领导专司其职、全力以赴，层层实行外经贸工作目标责任制，严格考核，兑现奖惩。进一步优化投资环境，在济宁高新区及省级开发区建立高效能管理、全方位服务的运行机制。支持海关、检验检疫等部门全面推进“大通关”建设，提升通关效率和服务功能。

　　突出四项重点。一是强化企业招商主体作用，增强企业吸纳外资载体功能，支持重点企业“以大引大”。二是把日韩、港澳台及东南亚、欧美和国内沿海地区作为招商引资的重点区域，卓有成效地举办专项经贸洽谈活动。三是围绕高新技术产业、资源开发、加工制造、农业产业化、服务业、旅游业和基础设施建设等重点领域，有针对性地开展对接招商。四是各县市区和济宁高新区筛选一批重点项目，组织专门班子运作推介，引进一批基地型、龙头型、科技型外资大项目。

　　创新五种方式。一是坚持完善以商招商。借鉴创办山推国际事业园的经验，鼓励引导现有外资企业增资扩股，推介、引荐更多外商来我市投资发展。二是坚持完善专业招商。选聘一批精通外语、熟悉国际经贸规则、掌握商务谈判技巧的高层次人才充实专业招商队伍，深入重点国家和地区开展职业招商。三是坚持完善网络招商。不断扩充“孔子经贸网”内容，完善功能，打造招商引资的重要平台。四是坚持完善代理招商。通过建立机制、落实待遇等措施，调动中介机构和代理的招商积极性。五是坚持完善会展招商。按照“政府搭台、市场运作、企业唱戏、扩大合作”的思路，创新办节方式，增强国际孔子文化节和中国专利高新技术产品博览会品牌效应，扩大经贸成果。

　　大力发展对外贸易。坚持经贸结合、以经促贸，创办济宁高新区出口加工贸易区。扶持出口创汇骨干企业。积极实施“走出去”战略，鼓励和支持有条件的企业到国外投资办厂或开展加工贸易，发展对外工程承包，带动技术、设备、产品出口和境外劳务输出。调整出口产品结构，提高机电产品特别是高新技术产品出口比重，按照国际标准化要求抓好农产品、畜产品、水产品出口基地建设，有效应对国际贸易壁垒，保持对外贸易持续快速增长。

　　（二）深化市场取向改革，实现体制机制建设新突破。按照十六届三中全会确定的各项改革任务，深入开展调查研究，认真制定实施方案，力求在发展混合所有制经济、推进社会事业改革、健全市场体系等方面取得新成效。

　　深化企业改革。一是规范完善企业改制行为。引导已改制企业健全法人治理结构，形成决策机构、监督机构与经营管理者之间的制衡机制，加快建立归属清晰、权责明确、保护严格、流转顺畅的现代产权制度。坚持改制先抓班子，对未改制企业，要选配德才兼备，有社会责任感和经营管理能力的领导班子。引导大型企业与科技研发水平高、市场网络健全、资金实力雄厚、综合竞争力强的国内外战略投资者发展合资合作。二是搞好企业内部改革。完善劳动用工和收入分配制度，促使企业转换机制，增强活力。三是大力培植上市企业资源。精心做好凯赛生物、中银电化、雪花生物化工、银河胶带、华仙股份、晨阳碳素等企业上市辅导工作，支持帮助具备条件的企业尽快上市。四是健全完善国有资产监管体制，确保国有资产不断增值。

　　加快社会事业改革。分类推进事业单位综合配套改革，重点搞好科研、教育、卫生、文化和市政公用事业等项改革，努力实现公用事业的社会化投资、市场化运营、产业化发展。

　　整顿规范市场经济秩序。抓好直接关系人民群众身体健康的食品、药品专项整治，开展烟草、文化、旅游市场整顿，加大技术监督和工商管理力度，严厉打击制售假冒伪劣商品、商业欺诈等违法犯罪活动。规范发展生产要素市场，培育产权交易市场，构建竞争、有序、公平、公正的开放市场体系。

　　（三）以增加农民收入为目标，实现农业结构调整新突破。立足统筹城乡发展、解决“三农”问题，着力拓展农业结构调整的深度和广度，促进农业增效、农民增收。

　　拉长“五大产业链”。围绕拉长畜牧产业链，积极引导专业镇、村和专业户扩大饲养规模。健全畜禽检疫监测体系，启动建设无规定疫病区，搞好优质品种和先进技术引进推广，全市畜牧业产值占农业总产值比重提高2个百分点。抓好渔湖产品精深加工，全市水产养殖面积达到65万亩。围绕拉长林果产业链，全面实施林业“六大工程”，年内全市完成封山育林10万亩，发展苗木花卉基地20万亩，干鲜果基地15万亩，速生丰产用材林基地35万亩，营造绿色通道800公里。围绕拉长瓜菜产业链，加快开发引进名优新品种，发展无公害蔬菜和有机农产品，年内瓜菜面积扩大到230万亩，建成高档特菜基地60万亩。围绕拉长粮棉油产业链，在确保粮食安全的前提下，突出抓好良种良法推广应用，提高单位面积产量和效益。围绕拉长劳务经济产业链，逐级建立完善劳务输出服务机构，定向开发一批劳务输出基地，分层次抓好劳动力技能培训，有针对性地开展对接输出，在全市组织实施“千人出境，40万人出省，60万人出市，100万人转移”的劳务输出活动，力求输出一人、致富一家，输出一批、致富一方。

　　进一步增加农业投入。提高财政支农比重，加大信贷支农力度，今年新增的教育、卫生、文化等事业经费主要用于农村。加强水利基础设施建设，高质量完成以田间排涝、河道疏浚、机井配套、小流域治理为重点的农田水利建设项目。加强乡村公路管理与维护，年内硬化农村公路2300公里。抓好农村饮水解困工程建设，解决20万人安全饮水问题。

　　推进农村各项改革。深化粮食流通体制改革，落实对农民直接补贴政策。取消农业特产税，逐步降低农业税率。稳定农村土地承包关系，引导规范土地经营权合理流转，搞好失地农民生活保障试点。积极发展农村经济协会和合作经济组织。加快发展农村二三产业，壮大集体经济实力。

　　（四）大力培植主导产业，实现工业经济新突破。按照新型工业化的要求，坚持优势传统产业与高新技术产业有机融合、双向促进，尽快构筑起以高新技术为先导、主导产业为支撑、骨干企业为主体的工业经济新高地。

　　培植支柱产业。煤电化产业，搞好煤炭精深加工，大力发展非煤产业，加快建设煤电化基地。生物技术产业，依托凯赛生物长链二元酸等重点项目，建设生物技术产业基地。机械制造产业，围绕推土机、挖掘机、专用汽车、内河船舶等产品，发展专业化协作，增强产业配套能力。医药食品产业，重点抓好抗生素系列产品开发与扩能，培植壮大医药生产基地；从实施农业标准化生产、扶持产业化龙头企业入手，加快建设绿色食品加工基地。纺织服装产业，培育打造高档服装品牌，改造提升纺织、面料、印染工艺装备及技术水平，拉长服装产业链条。新型建材及造纸产业，突出开发干法水泥、墙体材料等建材产品，加快推进林纸一体化。

　　开发高新技术产品。实施“名牌带动”战略，依托企业研发中心，运用高新技术和先进适用技术改造提升传统产品档次；立足生物技术、电子信息、机电一体化和新材料等重点领域，加快开发高新技术产品。今年全市高新技术产业产值占规模以上工业总产值比重达到19%。

　　壮大骨干企业。在搞好规划、科学定位的基础上，强化分类指导，通过外资嫁接、强强联合、上市融资等途径，推动有效资产向优势产业、优良企业、优秀企业家聚集。集中培植一批主业突出、核心竞争力强、规模效益高的大型企业集团。年内全市新增规模以上工业企业300家，年利税过千万元的企业达到130家，销售收入过10亿元的达到20家。

　　（五）加快发展服务业特别是旅游业，实现第三产业新突破。坚持改造传统服务业与发展新兴服务业并重，整合流通资源，拓展经营领域，创新营销方式，激发服务业特别是旅游业发展活力。

　　加快培植旅游产业。依法理顺旅游管理体制，正确处理开发利用与有效保护的关系，推进旅游资源的整合开发。精心策划“五圣之旅”精品旅游线路，抓好沿线重要景点开发建设，实现孔孟文化旅游景区的资源共享与联动；做活水资源文章，搞好运河文化带、北湖风景区整体开发；积极开发森林游、民俗游、农业观光游、休闲度假游等特色旅游，实现与传统旅游产品的互补与延伸。强化宣传促销和市场开拓，广泛吸纳国内外客源。办好世界旅游日中国主会场活动。配套完善旅游基础设施，协调推进“吃、住、行、游、购、娱”六大要素建设，在曲阜和济宁高新区规划建设旅游饭店，改善接待条件，丰富旅游产品，优化服务环境，延长游客游览时间，提高旅游业综合效益。

　　努力搞活商贸流通。走好外资活店、品牌兴店的路子，吸引更多的国内外知名商业品牌来我市创业发展。走好改制重组、盘活存量的路子，积极引进外资和民资参股控股，促使国合商贸企业转换机制、增强活力。走好创新经营、拓宽领域的路子，大力发展超市、连锁、配送等新型流通业态，兴办中介、信息、保险、社区服务、物业管理等新型三产项目，规划建设现代物流中心。支持办好“放心早餐”工程。发挥供销社点多面广的优势，构建起为农民生产生活服务的消费品流通网络、农资购销网络和农副产品购销网络，激活农村消费市场，促进农村二三产业发展。

　　重视财税金融工作。努力培植壮大财源，完善税收征管手段，健全税源控管体系，确保各类税收及时足额入库。调整优化财政收入和支出结构，不断提高可用财力，实现财政收支平衡。加快预算编制改革。深入研究财税政策，广开财源，增加财力。协调支持各金融机构认真落实稳健的货币政策，保持信贷投放的稳定性和适度增长。继续推进金融安全区创建和信用乡镇、信用企业、信用户评选活动，形成以道德为支撑、产权为基础、法律为保障的社会信用制度。

　　（六）持续扩大投资规模，实现产业项目建设新突破。坚持以招商引项目，以项目带投资，保持投资对经济增长的强劲拉动。通过抓好配套衔接尽快竣工投产一批，集中力量保证沃尔玛服装等500余个接转续建项目顺利建设，力争年内竣工投产率达到70%。通过落实包保责任加快建设一批，组成专门班子搞好跟踪服务，确保卡磨纺等169个重大产业项目完成年度建设任务。通过扩大招商引资筛选储备一批，选择符合产业政策导向、发展前景广阔的好项目积极申报立项，挤进国家、省重点建设“大盘子”。同时加强银企联手，搞好项目对接，加大金融对经济发展的支持力度。鼓励民间资本积极参与基础设施、公用事业建设。

　　（七）发展壮大县域经济，实现民营经济新突破。按照统筹城乡发展的要求，创造性地走好农业稳县、工业立县、三产活县、科教兴县、开放强县的路子，增强县域经济活力和竞争力，形成强市率先发展、中间县加速崛起、欠发达县跨越式赶上的新局面。

　　大力实施促强扶弱。用足用好激励扶持政策。邹城、兖州、曲阜三市要在更高起点上抓好结构调整，培植“立市”企业，提升产业层次，实现晋档进位。采取领导联系、对口帮扶、政策倾斜等措施，扶持欠发达县发挥比较优势，做强特色经济，尽快缩小差距。支持“双30”乡镇（30个经济强乡镇、30个经济薄弱乡镇）跨越发展，增强乡镇经济整体实力。

　　放手发展民营经济。把民营经济与招商引资、企业改革、结构调整、农业产业化扭在一起抓，拓宽经营领域，扩张发展规模。进一步清理废除不合理规定，降低市场准入门槛，在投融资、税收和对外贸易等方面一视同仁。用好用活扶持民营经济发展专项资金，建立健全中小企业信贷担保体系，积极为民营企业提供人才、技术、信息、法律服务。引导民营企业苦练内功，活化机制，注重资本运作，提高综合素质。继续实施“十百千”工程，支持科技型、外向型民营企业做大做强。今年全市民营经济总户数增长20%，注册资金、营业收入、上缴税金均增长35%。

　　（八）坚持“三高”要求，实现城市建设新突破。坚持新区开发与老城改造并举，基础建设与产业发展互促，完善功能与强化管理齐抓，加快建设组群结构大城市。

　　高起点规划。充分发挥规划的龙头作用，编制完善济兖邹曲重点建设区域控制性规划，提高详规覆盖率。放开规划设计市场，引进高层次规划设计人才，尽快完成一批规划设计精品项目。依据《济宁市城市发展总体规划》要求，进一步修订完善7县县城建设规划和小城镇规划，重点抓好22个中心镇建设。强化乡村规划指导。严格规划执法，纠正违规行为。

　　高标准建设。济宁高新区要进一步完善基础设施和承载功能，重点引进建设“高大外”产业项目，今年实现技工贸总收入300亿元、增长50%以上。指导邹城、兖州、曲阜三市依据功能分区定位，培植主导产业，走好“产业兴城、组团发展”的路子。济微线济宁段改造工程年内竣工通车。深入推进“绿亮清”二期工程建设。按照水林路统筹规划原则，启动北湖风景区开发。以南池水景园为重点推进老城改造。抓好经济适用房和廉租房建设。规划建设好市区主要出入口，切实解决城乡结合部和乡镇驻地脏、乱、差问题。抓好市区主要河道整治改造。继续维修、改造部分小街巷，加快供水、供气管网建设，扩大城市集中供热范围。

　　高效能管理。围绕天蓝、水碧、地绿、宁静、空气清新、环境优美、人居舒适的目标，进一步强化以治理大气、水环境和噪声污染为重点的城市环境综合整治，最大限度地消除大气异味和烟尘污染。大力推进城市管理综合执法，建立城市管理长效机制。重视做好经营城市工作。

　　四、立党为公，执政为民，实现和维护好人民群众的根本利益

　　各级政府要以为人民服务为天职，从解决实际问题入手，切实做好与人民利益密切相关的各项工作，实现人的全面发展。

　　努力扩大就业。就业是民生之本。把充分就业纳入经济社会发展总体目标，立足加快经济发展，不断扩充就业容量。今年全市新增城镇就业6万人，其中市直1.64万人；下岗失业人员再就业1.6万人，其中市直4000人。落实提供小额贷款、社会保险补贴等项政策，搞好困难群体特别是“4050”人员就业援助，确保夫妻双下岗、一户两代失业家庭中至少一人实现再就业。健全完善就业再就业服务体系，强化就业指导、培训，支持下岗失业人员自主创业、自谋职业。依法保障劳动者合法权益。

　　健全社会保障体系。社保是民生之基。深化养老、失业、医疗和工伤保险制度改革，进一步扩大社保覆盖面。依法将各类企业纳入社会保险范围，加大基金征缴和清欠力度。稳步推进下岗保障向失业保险并轨，搞好“三条保障线”衔接，切实保障下岗失业人员基本生活。摸清低保底数，增加低保投入，提高低保补助标准，实现动态管理下的应保尽保。年内继续办好10件实事。关心支持妇女儿童、残疾人事业，重视做好老龄工作。全面落实安全生产防范整治措施，减少各类事故发生，确保人民群众生命财产安全。

　　提高群众健康水平。健康是民生之需。以创建公共卫生安全区为抓手，健全疫情监测网络和信息报告制度，加强疾病预防控制体系和突发公共卫生事件救治体系建设，理顺规范120急救协调机制，提高公共卫生服务水平和应急能力。实行城乡医疗单位对口支援，加强乡镇卫生院建设，扩大农村新型合作医疗试点范围，建立贫困农民医疗救助制度。强化医疗市场监督管理。开展爱国卫生运动，继续做好农村改水改厕和垃圾填埋工作。发展振兴中医药事业。继续实施《全民健身计划纲要》，积极备战21届省运会，提高竞技体育水平。

　　五、统筹发展，协调推进，实现各项社会事业全面进步

　　坚持全面、协调、可持续的科学发展观，在加快经济建设的同时，重视发展各项社会事业，促进物质文明与政治文明、精神文明共同进步，经济与人口、资源、环境协调发展。

　　实施“科教兴市”战略。推进科技进步与创新。健全以企业为主体的科技创新体系，密切产学研结合，加强对关键技术和领域的科研攻关，提高自主创新能力。培植200家高新技术企业和100家中小型科技示范企业。发展科技中介服务组织，加速科技成果向现实生产力转化。加强知识产权保护和科技知识普及。做好气象和防震减灾工作。优先发展教育事业，巩固提高“普九”成果，完善“以县为主”的义务教育管理体制，消除校舍危房，严格控制学生辍学；进一步扩大高中阶段办学规模，提高办学质量和水平；调整职业教育、成人教育专业设置，着力培养实用型人才。建设办好济宁学院。全力支持曲阜师大、济宁医学院加快发展，充分利用和发挥好驻济高校及科研院所的人才、技术和创新优势。鼓励社会力量办学。

　　重视加强人才工作。牢固树立“人才强市”观念，把握培养、引进、使用三个关键环节，加快建设高素质党政人才、高绩效经营管理人才和高层次专业技术人才三支队伍。树立人人都能成才的观念，创新人才开发机制，多渠道、多层次、大规模开展人才培训，推进人才资源市场化配置。落实知识、技术、管理等要素参与收益分配的政策规定，改善高层次人才工作生活条件，营造留住人才、用好人才的良好环境。做好外事、侨务工作。办好留学生创业园和“百名人才”创业园，积极引进国外智力。

　　繁荣发展先进文化。继续推进文明社区、文明行业、文明村镇、文明单位等群众性精神文明创建活动，促进市民素质与城市文明程度同步提高。创作推出一批优秀文艺作品。深化文化体制改革，促进文化产业发展。加快广播电视、新闻出版事业发展。完善档案、史志、文博社会服务功能。

　　加快依法治市进程。发展基层民主，完善村规民约，落实村民自治和居民自治制度，规范政务、厂务和村务公开，保证人民群众依法行使民主权利。继续开展“四五”普法教育，增强全民法制意识。搞好法律服务与援助，依法保障弱势群体的合法权益。自觉接受市人大及其常委会的监督，支持人民政协履行职责，加强与各民主党派、工商联、无党派人士的联系，为工会、共青团、妇联、科协等人民团体发挥作用创造条件。认真办理人大代表建议和政协委员提案。围绕打造“平安济宁”，强化社会治安防控体系建设，持续开展严打整治斗争，坚决扫除“黄赌毒”等社会丑恶现象。健全完善矛盾纠纷排查调处机制，及时化解人民内部矛盾，妥善处置群体性事件。加强国防教育、国防动员、民兵预备役和人防工作，巩固发展“双拥共建”成果，关心支持中国人民解放军驻济部队和武警部队建设。做好民族、宗教、对台工作。增强国家安全意识，严厉打击“法轮功”邪教组织及敌对势力的破坏活动，维护社会政治稳定。

　　全面落实“三大国策”。积极推进计划生育综合改革和优质服务，加强流动人口等特殊人群计划生育管理，确保完成年度人口与计划生育责任目标。实行最严格的耕地保护政策，依法审批建设用地，切实保护耕地，抓好采煤塌陷地治理与土地复垦，确保耕地占补平衡。坚持资源开发与保护并重，坚决制止超设计能力、掠夺性开采煤炭行为，提高矿产资源开发利用水平。实施生态市建设工程，按照南水北调水质安全要求，加快沿线污水处理厂建设，强化重点流域和行业污染防治，确保实现达标排放。逐个企业落实脱硫除尘措施，实现二氧化硫和粉尘达标排放。推行清洁生产，发展循环经济，努力从源头上控制污染。加快自然保护区、生态示范区、生态功能保护区建设，构建人与自然和谐发展的生态环境。

　　六、牢记宗旨，从严治政，建设“为民、务实、清廉”人民政府

　　面对新形势新任务，各级政府及其工作人员必须紧紧抓住“第一要务”，时刻牢记“两个务必”，忠实践行“三个代表”，更好地履行管理社会、促进发展、服务人民的职责。

　　增强执政创业能力。深入学习邓小平理论和“三个代表”重要思想，全面贯彻党的十六大及十六届三中全会精神，认真钻研法律、经贸、科技、金融和管理等方面的知识，切实增强五种能力。一是增强科学判断形势的能力。总揽全局，遵循规律，把握本质主流，正视矛盾问题，力求站高一点、看远一步、想深一层，审时度势，据情善断，始终把握工作的主动权。二是增强驾驭市场经济的能力。坚持解放思想、实事求是、与时俱进，把加快发展的热情与科学求实的态度结合起来，把长远规划与分步实施结合起来，把大胆创新与遵纪守法结合起来，勇于探索实践，增创体制机制新优势，始终保持经济发展的旺盛活力。三是增强应对复杂局面的能力。学习掌握政策，深入调查研究，吃透上情，摸准下情，制定切实可行措施，妥善处置、及时化解各种矛盾问题，有效防止群体性事件和公共突发事件，做到既对上级负责又对群众负责，既对现实负责又对长远负责，既对局部负责又对全局负责。四是增强总揽全局的能力。善于谋全局、管大事，坚持统筹城乡发展，统筹区域经济发展，统筹经济社会发展，统筹人与自然和谐发展，统筹国内发展和对外开放。五是增强解决实际问题的能力。针对改革发展的瓶颈制约和人民群众的愿望要求，找准矛盾症结，落实相应举措，一个环节一个环节地突破，一个问题一个问题地解决，确保改革开放和结构调整顺利推进，维护、实现和发展好人民群众的根本利益。

　　提高依法行政水平。自觉学法、懂法、用法，全面掌握本职工作必备的各项法律法规，善于运用法律手段解决经济社会发展中的矛盾问题。以实施《行政许可法》为契机，加紧清理规范行政许可事项、许可主体，办好行政审批服务中心，不断提高审批效率和服务质量。完善重大问题议事规则和决策程序，建立人民建议征集和重大事项社会公示、社会听证、专家咨询等制度，提高决策的透明度和公众参与度。建立完善权责明确、行为规范、监督有效、保障有力的行政执法体制，加强行政执法队伍建设，进一步落实执法责任制和过错追究制，做到严格执法、公正执法、文明执法。

　　倡导求真务实作风。树立正确的政绩观，坚持讲实话、出实招、办实事、务实效，大力弘扬敢抓敢管、敢于负责、敢为人先的求真务实精神。卓有成效开展“工作落实年”活动，对各项工作落实、落实、再落实。进一步完善决策目标、执行责任、考核监督“三个体系”，分解任务，明确责任，加大对重要工作、重点事项督查落实力度，实行跟踪调度，严格考核奖惩。精简会议和文件，减少不必要的应酬，集中精力深入基层，调查研究，靠前指导，解决问题。力戒官僚主义、形式主义，坚决制止欺上瞒下、弄虚作假行为。强化行政效能监察，实行首问负责制、服务承诺制和限时办结制，提倡急事急办、特事特办。整治各种“三乱”行为，为企业生产经营、重点项目建设和各类投资主体提供优质服务，营造良好环境。

　　树立清正廉洁形象。保持发扬谦虚谨慎、不骄不躁和艰苦奋斗的作风，自重、自省、自警、自励，筑牢拒腐防变的思想道德防线。认真执行廉洁自律各项规定，进一步规范从政行为，做到堂堂正正做人，清清白白从政，扎扎实实办事。全面落实党风廉政建设责任制，强化责任追究。深入开展反腐败斗争，依法查处违法违纪大案要案。继续纠正部门和行业不正之风，解决群众反映强烈的突出问题。坚持抓源治本，加强审计监督，从源头上预防和治理腐败，以廉洁勤政的良好形象，赢得人民群众的信赖和支持。

　　各位代表！时代赋予我们重任，人民寄予我们厚望。让我们在中共济宁市委的坚强领导下，高举邓小平理论和“三个代表”重要思想伟大旗帜，解放思想，干事创业，比学赶超，加快发展，在新一年里创造更加辉煌的业绩，夺取改革开放和现代化建设的新胜利！
